# Extraction-free clinical detection of SARS-CoV-2 virus from saline gargle samples using Hamilton STARlet liquid handler

**DOI:** 10.1038/s41598-023-30993-2

**Published:** 2023-03-14

**Authors:** Vijay J. Gadkar, David M. Goldfarb, Ghada N. Al-Rawahi, Jocelyn A. Srigley, Duane E. Smailus, Robin J. N. Coope, Stephen Pleasance, Nicole Watson, Tammy Chen, Sunny Lam, Linda Hoang, Peter A. G. Tilley

**Affiliations:** 1 Department of Pathology and Laboratory Medicine, Division of Microbiology, Virology and Infection Control, BC Children’s and Women’s Hospital + Sunny Health Center, Vancouver, Canada; 2 Department of Pathology and Laboratory Medicine, Faculty of Medicine, University of British Columbia, Vancouver, Canada; 3 Canada’s Michael Smith Genome Science Centre at BC Cancer, BC Cancer Research Institute, Vancouver, BC Canada; 4 Public Health Laboratory, British Columbia Centre for Disease Control, Vancouver, Canada; 5 Department of Pathology and Laboratory Medicine, Division of Microbiology, Virology and Infection Control, BC Children’s and Women’s Hospital, Room No 2K9, 4500 Oak St, Vancouver, V6H 3N1 Canada

**Keywords:** Laboratory techniques and procedures, Microbiology, Infectious-disease diagnostics, Virology

## Abstract

As part of the COVID-19 pandemic, clinical laboratories have been faced with massive increases in testing, resulting in sample collection systems, reagent, and staff shortages. We utilized self-collected saline gargle samples to optimize high throughput SARS-CoV-2 multiplex polymerase chain reaction (PCR) testing in order to minimize cost and technologist time. This was achieved through elimination of nucleic acid extraction and automation of sample handling on a widely available robotic liquid handler, Hamilton STARlet. A customized barcode scanning script for reading the sample ID by the Hamilton STARlet’s software system was developed to allow primary tube sampling. Use of pre-frozen SARS-CoV-2 assay reaction mixtures reduced assay setup time. In both validation and live testing, the assay produced no false positive or false negative results. Of the 1060 samples tested during validation, 3.6% (39/1060) of samples required retesting as they were either single gene positive, had internal control failure or liquid aspiration error. Although the overall turnaround time was only slightly faster in the automated workflow (185 min vs 200 min), there was a 76% reduction in hands-on time, potentially reducing staff fatigue and burnout. This described process from sample self-collection to automated direct PCR testing significantly reduces the total burden on healthcare systems in terms of human resources and reagent requirements.

## Introduction

Collection of nasopharyngeal swab specimen (NPS) is still considered the gold standard for diagnostic detection of SARS-CoV-2^1,2^. This mode of sample collection has drawbacks, such as discomfort to the patient, and being resource intensive, as it requires a dedicated health care worker (HCW) to accurately collect or oversee the sample collection process. Poor performance of NPS sampling can result in false-negative results due to inadequate/improper sample collection. To address this, alternative sample types that are easy to administer, preferably by self-collection, were actively sought during the COVID-19 pandemic. Saliva was the first non-invasive, non-NPS sample type validated and found to have performance similar to the gold standard NPS specimen type^3–6^. The empirical observation that the SARS-CoV-2 virus is stable for > 72 h in this matrix^7^, and therefore does not require specialized collection devices or preservatives, opened up possibilities of performing at-home, self-administered sample collection. The development of “extraction-free” PCR protocols^8,9^ whereby the saliva is directly added to the RT-qPCR assay reaction mixture without extensive RNA purification processing, further enhanced the clinical acceptability of this sample type^5^. This led to Federal Drug Administration (FDA) approving saliva samples and a direct-PCR protocol for saliva testing, called the Saliva Direct^10^, for SARS-CoV-2 detection under emergency use authorization (EUA) regulations.

Notwithstanding the benefits of saliva as a simplified alternative to NPS sampling, its practical implementation for high-throughput SARS-CoV-2 detection has proven challenging for diagnostic laboratories^11^. Crude saliva is viscous and can congeal shortly after collection^6^. This makes it difficult to pipette manually or on an automated liquid handler. Discrete steps are required to reduce its viscosity, which include dilution in specific buffers^4,12,13^ or incubation with Proteinase-K^5^, followed by heat treatment (95 °C for 30 min), to inactivate the deproteinizing agent. These pre-analytical steps, though simple to implement, have to be performed manually, thereby imposing additional workload burden on a clinical laboratory. To alleviate this, collection of saliva in a liquid medium like universal/viral transport media (UTM/VTM) has been suggested^14^. However, the inhibitory nature of these liquefaction/stabilizing agents makes it difficult to implement the original Saliva Direct protocol, forcing diagnostic laboratories to process saliva samples using standard RNA purification methods^15–17^. This testing pathway is not only time consuming but contrary to the original goals of the Saliva Direct protocol which sought to make SARS-CoV-2 testing affordable, especially for resource challenged settings.

In latter half of 2020, two groups^18,19^ independently reported the use of saline mouth rinse/washes (saline gargle) for detecting the presence of SARS-CoV-2. Henceforth referred to as saline gargle, the collection of this sample type could be done without the need for a dedicated HCW in both adults and children (typically > 4 years old) while preserving their performance when compared with HCW collected NPS^20,21^ This freed up clinical resources and saved NPS collection devices, which were in critically short supply at the time^22^. Further studies showed saline gargle was also amenable to extraction-free PCR, but unlike saliva, did not require complex pre-analytical processing due to its water-like consistency^23,24^. The simplicity of this process opened up the possibility of automating the testing process on a liquid handler. The goal of the present study was therefore to describe the optimization and implementation of the Spike/ORF8/RNaseP (SORP) multiplex PCR test to the gargle Direct-PCR (GDirect-PCR) format on an automated liquid handling platform—the Microlab STARlet (Hamilton Robotics, NV, USA) liquid handling system, henceforth referred to as Hamilton GDirect-PCR (HGDirect-PCR).

## Results

### GDirect-PCR using the SORP Triplex assay (version 1)

Initial testing on the feasibility of using the direct-PCR on saline gargle samples was done on a cohort of 38 positive and 75 negative SARS-CoV-2 samples using two assays – the N1/N2 US-CDC and SORP (version 1). The US-CDC’s Nucleocapsid assay detected the N1 targets in the 38 positive saline gargle samples using the GDirect-PCR method; however, four samples were falsely negative for the N2 target (sample no: FS5, FRS10, FRS16 and FRS18) (Table 1). When the same samples were tested using the standard RNA extraction-based protocol, no false-negative results were recorded. The mean increases in C_T_ value between the standard extracted RNA and direct-PCR approach for N1 and N2 gene targets were 2.74 and 5.74 respectively. The RNaseP internal control was detected in all positive and negative gargle samples in both standard and extraction-free methods.

**Table 1:** C_T_ values obtained for each of the gene targets using the reference assay (E/RdRp), SORP triplex and US-CDC’s N1/N2 assay.

		Type	Standard method				GDirect-PCR		Standard method		GDirect-PCR	
			Reference assay		SORP		SORP		N1/N2-US CDC		N1/N2-US CDC	
			RdRp	E gene	S	ORF8	S	ORF8	N1	N2	N1	N2
1	FS1	Frozen	31.17	31.56	30.82	32.68	34.51	34.72	29.91	30.53	34.35	37.66
2	FS2	Frozen	22.30	21.88	21.09	23.08	25.93	25.80	20.56	20.95	31.51	26.20
3	FS3	Frozen	28.28	28.49	27.73	29.99	31.17	33.01	27.62	27.77	30.99	33.35
4	FS4	Frozen	26.60	26.60	26.09	28.31	29.22	30.09	25.41	26.13	27.29	31.60
5	FS5	Frozen	24.67	24.89	23.80	29.53	28.19	31.45	23.30	24.89	26.37	**41.0**
6	FS6	Frozen	24.80	25.10	24.40	26.28	29.19	29.68	23.74	23.89	26.97	29.45
7	FS7	Frozen	26.90	27.21	25.87	26.30	30.46	30.77	25.20	26.96	28.10	30.80
8	FS8	Frozen	30.60	30.80	31.20	30.12	32.07	33.96	29.43	30.23	31.83	34.14
9	FS9	Frozen	28.98	29.38	27.89	28.45	30.46	32.26	28.20	29.28	30.05	33.19
10	FS10	Frozen	24.70	24.70	23.81	26.05	27.32	28.21	23.76	23.74	26.04	29.03
11	FS11	Frozen	28.20	28.90	27.71	29.75	33.98	32.52	26.75	27.16	30.02	34.10
12	FS12	Frozen	28.10	28.80	27.50	30.01	29.82	31.46	26.90	27.27	29.39	32.58
13	FS13	Frozen	25.92	26.61	25.10	27.35	30.18	29.99	24.54	25.36	27.23	30.90
14	FS14	Frozen	30.80	30.26	29.77	30.47	32.59	34.37	29.43	30.29	32.15	35.50
15	FS15	Frozen	32.21	32.66	31.76	33.34	36.36	36.77	25.52	25.63	33.87	38.74
16	FS16	Frozen	23.40	23.60	23.50	24.12	24.29	26.30	22.39	23.60	24.79	27.02
17	FS17	Frozen	22.64	23.27	21.89	23.49	27.90	27.08	20.42	20.92	24.61	27.29
18	FS18	Frozen	21.16	20.67	21.47	23.92	26.34	25.31	18.56	19.40	23.51	25.81
19	FS20	Frozen	27.90	27.80	27.17	29.33	30.17	30.83	28.51	27.87	28.30	31.73
20	FRS1	Prospective*	26.10	26.40	25.88	28.21	29.34	30.57	25.82	26.43	27.64	30.81
21	FRS2	Prospective*	26.60	27.50	26.23	28.31	32.23	31.73	26.66	26.77	30.55	33.54
22	FRS3	Prospective*	30.90	30.90	30.31	32.24	31.32	32.60	30.39	30.59	31.00	33.47
23	FRS4	Prospective*	31.60	31.70	30.56	32.18	33.95	34.93	30.67	30.91	33.22	36.66
24	FRS5	Prospective*	25.80	25.80	25.29	27.02	31.63	30.22	21.74	25.65	29.11	32.25
25	FRS6	Prospective*	24.50	24.50	23.89	25.79	26.22	26.95	24.15	24.00	25.60	28.25
26	FRS7	Prospective*	25.70	25.90	24.97	27.19	30.84	30.24	25.71	26.10	28.01	31.67
27	FRS8	Prospective*	26.20	26.90	25.83	27.96	28.47	30.38	25.47	26.80	28.19	31.11
28	FRS9	Prospective*	19.40	19.40	18.44	20.62	20.11	21.67	19.24	18.97	19.90	22.16
29	FRS10	Prospective*	30.70	31.60	30.38	33.81	34.03	36.44	31.71	34.65	34.56	**41.0**
30	FRS11	Prospective*	34.40	34.70	33.30	35.40	36.59	37.21	35.86	32.13	37.04	39.64
31	FRS12	Prospective*	31.60	32.10	31.23	33.24	36.65	36.36	31.94	30.91	33.52	37.07
32	FRS13	Prospective*	30.80	31.00	31.45	32.82	36.11	35.50	31.55	30.36	32.62	35.99
33	FRS14	Prospective*	31.30	31.60	30.87	32.51	37.38	36.06	30.45	31.43	33.63	38.81
34	FRS15	Prospective*	33.49	34.21	32.72	35.41	**41.0**	**41.0**	33.08	33.58	34.03	37.49
35	FRS16	Prospective*	33.61	32.67	35.18	38.46	38.7	37.78	36.86	38.09	36.49	41.0
36	FRS17	Prospective*	33.33	33.33	32.13	34.40	39.8	37.16	31.58	31.89	32.86	35.12
37	FRS18	Prospective*	35.11	36.05	34.28	35.87	**41.0**	38.99	34.26	33.75	34.20	**41.0**
38	FRS19	Prospective*	25.01	25.05	24.54	26.65	31.6	30.15	24.31	24.17	26.27	30.01
		Avg	**28.04**	**28.28**	**27.53**	**29.49**	**31.77**	**32.12**	**27.15**	**27.61**	**29.89**	**33.35**

Standard method: Extracted RNA method, GDirect-PCR: Extraction free method, C_T_ cut-off was kept at 40.

Undetermined target was assigned an arbitrary value of 41 (depicted in bold).

Prospective*: Samples did not undergo a freeze–thaw cycle.

When the same 38 positive saline gargle samples were tested on the SORP assay (version 1), using the standard RNA extraction protocol, both S and ORF8 gene targets were detected in all the samples, including the RNaseP internal control (Table 1). When tested by the GDirect-PCR, both the S and ORF8 were detected in all but two samples: FRS15 (Spike and ORF8 negative) and FRS18 (Spike-negative, ORF8-positive) (Table 1). The mean increases in C_T_ value between the standard extracted RNA and direct-PCR approach for S and ORF8 gene targets were 4.24 and 2.63 respectively. No false positives were detected amongst the 75 SARS-CoV-2-negative gargle samples tested with the GDirect-PCR protocol. This gave the GDirect-PCR process a positive percent agreement of 97.37% (95% CI 86.2–99.9%) and an overall agreement of 99.12% (95% CI 95.2–99.9%) with respect to the reference assay.

### Development of SORP version 2 triplex assay

When we applied the original SORP assay (version 1) on a cohort (n = 200) of SARS-CoV-2 positive saline gargle samples, we noticed two issues (a.) loss of the spike gene target in approximately 7% of the positive samples and (b.) strong RNaseP signal from clinical samples (C_T_ = 18–28). While the presence of large quantities of human cells in the saline gargle samples explains the strong RNaseP signal, the frequent dropout of the spike gene target was unexpected. When the same single target positive (S^-^/ORF8^+^) samples were re-tested using extracted RNA, positive amplification for the spike gene was consistently detected. Manipulation of the individual concentrations of the Spike-F1/-R1 primers and Spike-P1 probe did not correct the problem (data not shown).

**Figure 1:**
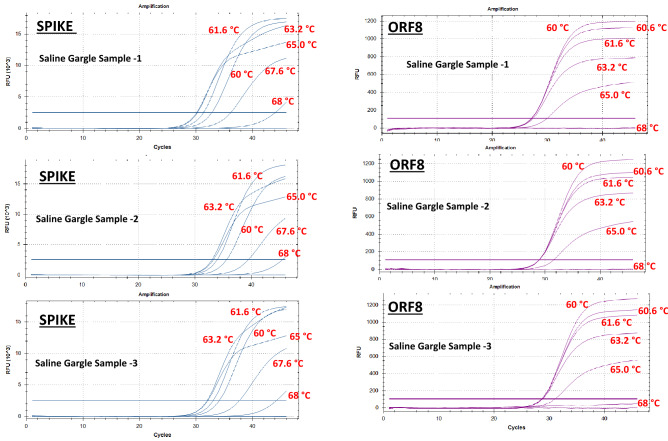
Representative example from three saline gargle samples tested using extraction-free PCR using SORP ver:1 assay. Variation in S and ORF8 gene signal intensity, at different annealing temperatures (60 °C to 68 °C) quantified in relative fluorescence units (RFU). As specified by the manufacturer of the Luna Probe One-Step RT-qPCR mix, 60 °C was considered as the optimal annealing temperature and used as reference temperature.

The optimal annealing temperature was determined using temperature gradient PCR (60 °C to 68 °C) on a cohort of positive saline gargle samples (Fig. 1). While the annealing temperature (60 °C to 63 °C) had no appreciable effect on the amplification profile of the ORF8 gene target, the spike gene showed optimal amplification at annealing temperatures of > 65 °C (Fig. 1). This suggested that the PCR annealing temperature of 60 °C, set in the original SORP version 1 assay for purified RNA templates, was not optimal for saline gargle templates used in direct-PCR conditions. When RNA was extracted from saline gargle samples and used as a purified template, both the S/ORF8 targets amplified optimally at 60 °C (Fig. 4_suppl) and any departure from this temperature (> 60 °C), resulted in a gradual decrease in the detection signal of both these targets. This suggested that the performance of the spike gene target was influenced by both the temperature and nature of input template (purified RNA vs unpurified saline gargle). The ORF8 amplification was not affected by the type of input template but an, increase in temperature from the optimal 60 °C did result in dampening of the amplification signal (Fig. 4_Suppl).

To address the sub-optimal performance of the spike gene target, we truncated the original spike forward primer Spike-F1^23^, resulting in its optimal amplification at 60 °C in the GDirect-PCR (Fig. 5_suppl). This new truncated spike primer, designated Spike-F1-M4, replaced the original Spike-F1 primer. Application of the new Spike-F1-M4 resulted in an optimal amplification of both the spike and ORF8 gene targets using an annealing temperature of 60 °C in GDirect-PCR.

The new low efficiency RNaseP R-3 primer resulted in a weaker signal (increase in an average 3–4 C_T_ values) in the human DNA control when tested on a cohort (n = 12) of negative gargle samples (Fig. 6_suppl). Use of the Spike-F1-M4 primer with the weaker RNaseP amplification primers improved the detection of both the S/ORF8 targets when tested on previously positive SARS-CoV-2 gargle samples (Fig. 7_suppl).

### Effect of heat inactivation on detection of SARS-CoV-2

**Figure 2:**
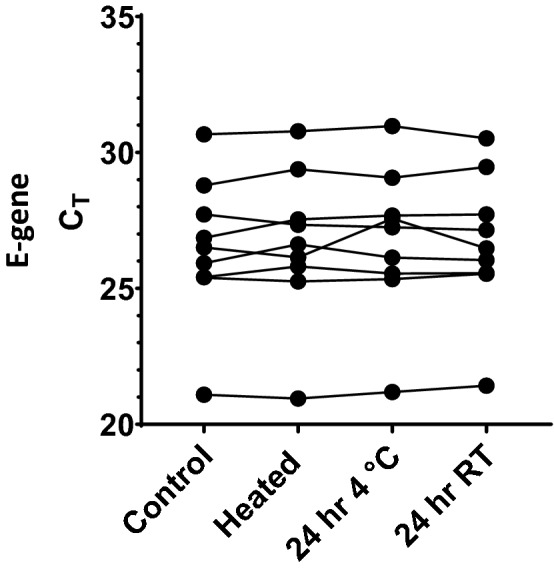
Variation of E-gene signal from saline gargle samples tested for SARS-CoV-2 after heat inactivation (65 °C for 60 min). Aliquot of the heated sample was stored at 4 °C and room temperature (RT), for 24-h, prior to E-gene detection. Control = original sample tested immediately post-heat inactivation.

Heat inactivation of the saline gargle samples at 65 °C for 1 h did not result in a significant loss in the E-gene signal (Fig. 2). The mean C_T_ change was ΔC_T_ =  + 0.16 pre- and post-heating. The mean ΔC_T_ was − 0.26 and − 0.17 when the same cohort of heated samples was stored at 4 °C and room temperature for 24 h, respectively, prior to testing.

### Performance assessment of the Hamilton STARlet for direct-PCR

In a 96-well plate run containing 92 replicate samples, the average C_T_ values of the Spike and ORF8 gene targets were 28.56 (SD = 0.10) and 29.35 (SD = 0.10) respectively. The coefficient of variation of the C_T_ values for both the Spike and ORF8 gene targets was 0.35%. No cross-contamination was detected in the checkerboard-dispensing scheme.

### Clinical validation of HGDirect-PCR workflow

**Figure 3:**
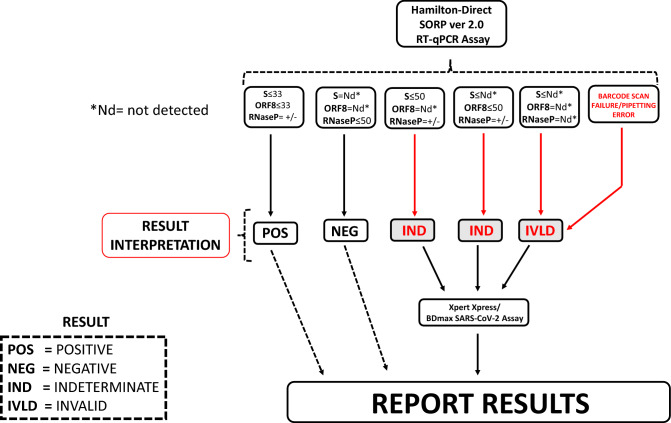
Interpretation algorithm for HGDirect-PCR workflow. Samples returning either IND (Indeterminate) or IVLD (Invalid) result were submitted for retesting on either the XpertXpress or BDMax SARS-CoV-2 Assay.

In the first phase of validation, a total of 908 saline gargle clinical samples were tested in parallel on the reference assay and on the HGDirect-PCR workflow over 10 days. The results of the HGDirect-PCR results were recorded and interpreted using an algorithm as outlined in Fig. 3. The HGDirect-PCR workflow detected 78 positives (S^+^/ORF8^+^) from the total of 87 positives detected by the reference assay (Table 2). Of the remaining 9 positives, 6 samples were positive for single gene target (S or ORF8), making them “indeterminate” (“IND”), whereas 3 samples did not return a result, due to liquid dispensing failure on the Hamilton Starlet Instrument (“IVLD”) (Table 2). Of the 821 samples which tested negative on the reference assay, 793 were classified as negative (S^-^/ORF^-^/RNaseP^+^) by the HGDirect-PCR workflow (Table 2). The remaining 28 samples returned with a discordant result: 11 single gene positives (“IND”), 10 RNaseP failure (“IVLD”) and 7 liquid dispensing error (“IVLD”). Thirty-seven samples from the total 908 samples tested on the HGDirect-PCR workflow would have required retesting (Table 2) for result confirmation, for a retesting rate of 4.07% (37/908). No false positive or false negatives were recorded on the HGDirect-PCR workflow, giving this testing process a 100% sensitivity and specificity, with 95.9% detection accuracy provided that a confirmatory retesting of samples which returned with an IND/IVLD result was performed.

**Table 2:** Validation of the HGDirect-PCR workflow using 908 saline gargle samples, tested in parallel by the standard method (KingFisher) and the HGDirect-PCR workflow.

HGDirect-PCR	Standard method (KingFisher)		Total (n = 908)
	POS (n = 87)	NEG (n = 821)	
POS	78	0	78
IND + IVLD	9	28	37
NEG	0	793	793
Total	87	821	908

In the live application of HGDirect-PCR testing on 152 saline gargle samples, 8 positives were detected (8/152) and 144 negative (144/152), with 2 discordant results (Table 3). These two discordant results were single gene positive (“IND”) or RNaseP detection failure (“IVLD”), resulting in a test failure rate of 1.3%. These discordant results were immediately resolved on the Xpert Xpress SARS-CoV-2 assay and found to be negative.

**Table 3:** Result of live testing of 152 saline gargle samples on the HGDirect-PCR workflow.

HGDirect-PCR	Standard method (Kingfisher)		
	Positive	Negative	Total
Positive	8		8
Indeterminate		1^a^	1
Invalid		1^b^	1
Negative		142	142
Total	8	144	152

^a^E^+^/RdRp^-^ (confirmed Negative by Xpert Xpress).

^b^RNaseP fail (confirmed Negative by Xpert Xpress).

### Time/workflow audit of setting up of HGDirect-PCR vs standard PCR

**Figure 4:**
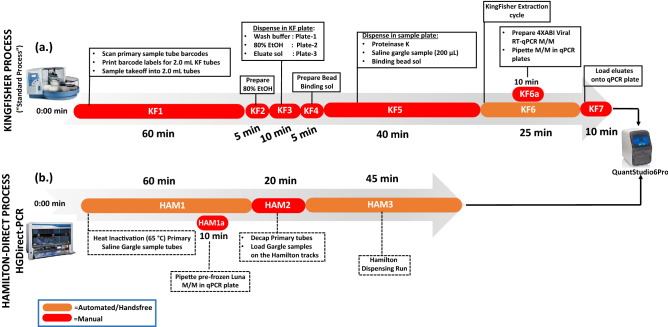
Setup time (min) required to process 91 saline gargle samples on the (**a**) KingFisher process (“standard process”) and (**b**) Hamilton-Direct process (“HGDirect-PCR”). Setup time (minus PCR cycling) required for (**a**) KingFisher process = 155 min (Hands on time = 130 min) and (**b**) HGDirect-PCR = 125 min (Hands on time = 30 min). Total time (setup + PCR cycling) for KingFisher and HGDirect-PCR is 200 min and 185 min respectively.

To process 91 saline gargle samples, up to the PCR cycling stage, it took the technologist an average of 125 min on the HGDirect-PCR workflow, of which only 30 min was the actual hands-on time (Fig. 4). In contrast, it took the technologist 155 min by the standard method, of which 130 min was the hands-on time (Fig. 4), to setup 91 samples. If the PCR cycling time was included, the standard process took 200 min whereas the HGDirect-PCR took 185 min to process a batch of 91 saline gargle samples.

## Discussion

Since COVID-19 was declared a worldwide pandemic by the WHO in early March 2020, diagnostic laboratories experienced unprecedented demands to scale up their testing capacity, resulting in staffing and reagent shortages. In order to increase lab efficiency, we developed a simpler direct PCR assay, removing the need for extraction, and automated the process to take advantage of primary tube testing. Previous reports have described Direct-PCR methods using manual methods, but these are repetitive, time-consuming and contribute to staff fatigue. Interestingly, few studies to date have described the benefits of implementing an automated liquid handler in an extraction-free PCR testing workflow. In one study by Blairon et al.^25^, the Nimbus extraction platform, part of the Allplex SARS-CoV-2 Assay RT-qPCR testing system (Seegene Technologies, Korea) was re-configured to perform a pre-dilution step of the clinical specimen, followed by dispensing into the assay mixture, resulting in both improved throughput (86.4 vs. 97.8%) and turnaround time (19:18 h vs. 09:03 h) over the manual process. While this study used the UTM sample type, we are not aware of any other study where a liquid handling system was used for extraction-free PCR processing on the saline gargle sample type.

An additional benefit of the automated process was the use of primary tube testing. In order to implement this, we made two changes in our workflow: (1.) unique barcode labels were attached to the tubes, which could be read by the Hamilton STARlet’s scanning system and (2.) we heat inactivated the specimens (65 °C for 60 min) in the primary collection tubes, so that they could be safely processed on the Hamilton’s open platform in a standard biosafety level 2 (BSL-2) laboratory. The lower 65 °C temperature at a longer duration was chosen due to concerns about the integrity of the primary sample collection tubes at 95 °C, which is faster but has an adverse effect on the downstream PCR signal^26,27^.

In our experience, the choice and optimization of the SARS-CoV-2 RT-qPCR multiplex assay was critical for the ultimate success of the extraction-free PCR process. This is because the SARS-CoV-2 RT-qPCR multiplex assays published to date have been developed for purified SARS-CoV-2 RNA template. Direct adoption of these multiplex assays in an extraction-free workflow can lead to sub-optimal amplification. For example, Vogel et al. (2020) noticed random dropout of the N2 SARS-CoV-2 gene target when the US-CDC’s authorized N1/N2 multiplex assay was used on saliva templates. As a result, the FDA-EUA licensed Saliva Direct protocol relies on a single SARS-CoV-2 N1 gene PCR (with human RNaseP as the sample control) for detection of SARS-CoV-2^10^ Similar observations have also been made in other extraction-free PCR studies where certain SARS-CoV-2 multiplex combinations have resulted in lower detection rates. For example, E/N1 gene targets in multiplex combination had a detection rate of ~ 70% as compared to ORF1ab/N1 or ORF1ab/N2, which had > 98% analytical sensitivity^26^. We also noticed poor performance of our original S/ORF8 assay where random dropout of the S gene target was observed on the GDirect-PCR workflow. However, this was addressed when the assay was re-optimized (SORP version 2). We would like to also add that, due to frequent mutations observed in the S-gene (https://covariants.org/), there could be possibilities of observing S-gene dropouts (S^-^/ORF8^+^). Getting this result would invariably lead to excessive burden of additional confirmatory testing as per our testing algorithm (Fig. 3). Under such circumstances, the most prudent remedial measure would be to perform a detailed *in-silico* analysis of the S-gene’s primer/probe set and implement any potential modification(s) to its sequence.

The gains in testing efficiency through the HGDirect-PCR process need to be balanced with the need for increased confirmatory testing. Of the 908 gargle samples tested during the HGDirect-PCR validation, 4.07% (37/908) samples required retesting.

This study does have limitations. We did not test NPS samples on our HGDirect-PCR system. This was because the Hamilton STARlet’s liquid handler pipette tips frequently collided with the swabs sticks still present in the primary sample tubes. Although one way to circumvent this would be to dispense the UTM/VTM liquid into a non-swab containing tube, this would have added an extra time-consuming and laborious step, which would remove much of the benefit of the automated process. An additional limitation of this study is the comparison of the existing manual extraction process to the automated direct-PCR process. An alternative would have been the automation of loading of samples into the standard extraction instrument, which was implemented upon the study completion. This had some additional gains through reduced handling time.

In summary, direct extraction-free PCR testing for SARS-CoV-2 detection can be performed from primary tubes on a liquid handler such as the Hamilton STARlet. With careful optimization, direct-PCR can be reliably implemented in an automated workflow, saving technologist time and potentially decreasing staff fatigue for high volume testing. The process described here may be of interest to clinical laboratories interested in incorporating a liquid handler for performing extraction-free SARS-CoV-2 PCR in high volumes.

## Methods

### Clinical specimens

Clinical specimens submitted for routine SARS-CoV-2 testing at the Microbiology & Virology Laboratories of BC Children’s Hospital (BCCH) were used. Samples were collected from symptomatic children and adults, including health care workers, at our onsite COVID-19 testing clinic, as well as at a community-based drive-through COVID-19 testing clinic. As described previously^18^, patients were directed to observe an educational video (http://y2u.be/V9xonNTtApY; http://y2u.be/ZvqjkbD-moA) for self-collection of the sample and were provided with the collection kit. Samples were forwarded to the laboratory within 1–6 h from the time of collection. This research was performed in accordance with the Declaration of Helsinki and the study was approved by the University of British Columbia Research Ethics Board (**H20-02538**). Residual samples were anonymized prior to use and a waiver of consent was granted for purposes of this assay validation study.

### SARS-CoV-2 real time PCR assay

#### Standard nucleic acid extraction & real-time PCR:

The primary SARS-CoV-2 assay used at the Microbiology & Virology laboratory was developed by the Public Health Laboratory at the British Columbia Centre for Disease Control (BCCDC). This assay targeted the SARS-CoV-2 RdRp and E-genes, with the human RNaseP as the internal control^28^. This assay, henceforth referred to as the “reference assay,” was used routinely to test clinical samples. The total nucleic acid (TNA) used for this assay was purified on the KingFisher Flex automated extraction instrument (ThermoFisher, Carlsbad, CA), using the MagMAX Viral Pathogen Nucleic Acid Isolation Kit (ThermoFisher) following the manufacturer’s recommendations. Five microliters of the final TNA eluate (50 µL), dispensed in the KingFisher 96-well plate, was transferred to the ABI plate for the RT-qPCR assay. This workflow, henceforth referred to as the “standard extraction process,” is summarized in Fig. 4.

### Gargle direct-PCR (GDirect-PCR)

Initial testing for extraction-free PCR was done on a cohort of 38 SARS-CoV-2 positive and 75 negative saline gargle samples, previously tested using the standard extraction assay. The samples were placed in a laboratory convection oven at 65 °C for 60 min for viral inactivation, then cooled at room temperature (RT) for 10 min. 5 µL of the primary gargle sample was then directly added manually into the RT-qPCR reaction. Two separate SARS-CoV-2 multiplex assays were used for testing: the Spike-ORF8 (SORP) triplex assay^23^ and the N1/N2 US-CDC’s Nucleocapsid Assay^2^.

The original SORP triplex PCR assay^23^ required modification prior to its implementation on the HGDirect-PCR. Specifically, the original Spike-F1 primer was truncated to give a new primer, Spike-F1-M4, and the mRNA RNaseP-targeting RNaseP-R8 reverse primer was replaced by a new primer RNaseP-R3 (see results above). The modified SORP assay is referred to as “SORP ver: 2” (Table 4), to distinguish it from the original SORP assay^23^, henceforth referred to as “SORP ver: 1” assay.

**Table 4:** Sequence of forward and reverse primers and TaqMan probes used for the SORP assay.

PRIMER/PROBE	Sequence	Nucleotide position	Ref
Spike-F1	CCACTAGTCTCTAGTCAGTGTGTTAATY	21,568–21,595	Gadkar et al.^23^ (SORP:ver1)
Spike-R1	AAACTGAGGATCTGAAAACTTTGTC	21,618–21,647	
Spike-P1	**FAM-**CAACCAGAA/**ZEN**/CTCAATTACCCCCTGCATACA-**IABlkFQ/**	21,690–21,716	
Spike-F1-M4	CACTAGTCTCTAGTCAGTGTGTTAAT		Modified from Spike-F1^23^ for HGDirect-PCR application (SORP:ver2)
ORF8-F1	GGAGCTAGAAAATCAGCACCTTTAA	28,041–28,065	Gadkar et al.^23^ (SORP:ver1)
ORF8-R	TCGATGTACTGAATGGGTGATTTAG	28,093–28,117	
ORF8-P	**Cy5**-TGAATTGTG/**TAO**/CSTGGATGAGGCTGG-**IABlkRQ**/	28,067–28,090	
RNaseP-F	AGATTTGGACCTGCGAGCG		US-CDC
RNaseP-P	**NED**-TTCTGACCTGAAGGCTC-**MGBNFQ**		BCCDC in house design (Tracy Lee) Modified from US-CDC for MGB chemistry
RNaseP-R	GAGCGGCTGTCTCCACAAGT		US-CDC
RNaseP-R3	TCTGGGAGACCTGACCG		Present work

Position of the Spike and ORF 8 primers and probes based on the alignment to the Wuhan-Hu-1 SARS-CoV-2 sequence (RefSeq:NC_045512.2).

The Direct-PCR was performed using the Luna Probe One-Step RT-qPCR mix (Cat #: E3006E; New England Bio labs, Whitby, ON). Five μL of the saline gargle sample was added to 25 μL of the assay reaction mix which consisted of: 12.5μL Luna probe mix (2X), 1.25μL Reverse Transcriptase (20X), Spike-F1-M4/R1 primers (0.4 μM each), Spike-P1 probe (0.2 μM), ORF8-F1/ORF8-R primers (0.4 μM), ORF8-P probe (0.2 μM), RNaseP-F/R3 primers (0.2 μM) and RNaseP-P probe (0.2 μM). The 96-well RT-qPCR reaction plate was vortexed (800 RPM for 1 min) on ABI Digital Vortex-Genie 2 Shaker (Cole Palmer, Montreal, QC) to mix the reaction components. The cycling conditions for direct-PCR were: 55 °C for 5 min, 60 °C for 5 min (Reverse Transcription), 95 °C × 60 s (Enzyme Activation) followed by 50 cycles of 95 °C at 10 s and 60 °C at 60 s. Thermocycling and fluorescence capture was performed on the QuantStudio 6Pro instrument (ThermoFisher). For operational efficiency, SARS-CoV-2 Luna RT-qPCR mastermix (containing S, ORF8 and RNaseP primers and probes, and the reverse transcriptase enzyme) was made beforehand in large batches. Each batch (pre-dispensed in 2 mL tubes sufficient for one 96-well plate) was QC’d and stored frozen at -80 °C.

### Modification of RNase P amplification—“low efficiency”

To avoid competition between the internal control PCR and viral PCRs in weakly positive clinical samples, a new reverse primer, RNaseP-R1, was designed approximately 89 bp downstream of the US-CDC’s RNaseP-R primer (Fig. 1_Suppl) in routine use in British Columbia. A truncated version of the RNaseP-R1 primer was custom synthesized by deleting six bases from its 3ʹ-end. This new primer, designated as RNaseP-R3 (Fig. 1_Suppl), was tested on a convenience sample, which consisted of a pool of saline gargle samples that previously tested negative for SARS-CoV-2 using the reference assay (E/RdRp/RNaseP).

### Heat stability studies

A cohort of 9 saline gargle samples, previously tested positive for SARS-CoV-2, were used for studying the effect of heat inactivation on the final RT-qPCR signal. Individual gargle samples were heat inactivated as described earlier. Aliquots were saved prior to heating, post-cooling, and following 24 h of storage at 4 °C and RT. The aliquots were submitted for RNA extraction and PCR for the SARS-CoV-2 E-gene^29^.

### Direct-PCR using the Hamilton STARlet liquid handling system

After logging in the patient samples using the SunQuest 6.4 system (Sunquest Information system, Tucson, AZ), barcode labels incorporating a unique specimen number were applied to the primary sample tubes (Fig. 2_Suppl). Batches of 91 tubes were then incubated at 65 °C for 60 min to inactivate the SARS-CoV-2 virus^26,30^ then cooled at room temperature for 10 min. Tube caps were then removed as the tubes were loaded into four 24-tube Hamilton racks (Fig. 2_Suppl). Two positive and negative controls were loaded in the last rack.

Tube barcodes were scanned at loading using the Hamilton STARlet’s onboard tube scanner. To handle potentially misapplied labels, a scanning method was written that flagged sample tubes that failed to scan, giving the operator the option to rotate and rescan the sample tube with a handheld scanner, or enter the specimen number at the keyboard. The method then wrote three Microsoft Excel files containing IDs and plate locations for (a.) all transfers (b.) all successful transfers and (c.) all failed transfers. A macro utility was built in Microsoft Excel 2016, to accept the sample barcode data and convert it into a 96-well PCR plate format to be imported into the QuantStudio 6Pro instrument’s Design & Analysis Software ver 2.6 (ThermoFisher). In our workflow, this Excel macro interface was pre-loaded on the PC computer, which controlled the QuantStudio 6Pro instrument.

Given the size of the primary tube, the transfer of 5 µl of saline gargle sample to the RT-qPCR reaction plate required two pipetting steps (Fig. 2_suppl). In the first step, 100 µl of the saline gargle sample was transferred into an intermediate plate (ABGene 1400 96-well plate; ThermoFisher) by a 1000 µl volume capacity pipette tip. After this transfer, 5 µL of saline gargle was dispensed from the intermediate plate into the RT-qPCR assay mixture using a 10 µl volume tip.

The first transfer used Hamilton’s *HighVolumeFilter_Glycerin80_DispenseSurface_Empty* liquid class with the following modifications: Liquid level sensing was by pressure only (set to high sensitivity) with capacitive sensing turned off to avoid triggering on surface bubbles. A 10 µl transport air gap was found optimal to prevent droplet formation without enabling air–liquid boundary inversion inside the tips. The 10 µl dispense used the same liquid class but with capacitive sensing on and a 2 µl transport air gap.

### Pre-validation testing

Prior to initiating validation of the extraction-free PCR assay using the Hamilton STARlet liquid handler, two quality parameters were assessed (1.) inter-well pipetting precision and (2.) cross-contamination assessment.

For inter-well pipetting precision, a pool of positive samples (C_T_: 18–20) was used. This sample was heat inactivated as described earlier and diluted in 200 mL of saline (0.9% NaCl), to give a final C_T_ of 27–29. Two mL aliquots of this diluted positive gargle sample were then manually dispensed into 92 primary gargle collection tubes having unique barcode labels and placed in the Hamilton STARlet’s sample racks. After dispensing of the sample (5µL) by the liquid handler into the SORP version 2 RT-qPCR reaction mixture, the PCR was run and the C_T_ values of the S/ORF8 signal were recorded from each of the 92 reactions. The mean C_T_, standard deviation and coefficient of variation were calculated as a measure of precision.

For cross contamination assessment, a “checkerboard” dispensing profile (Fig. 3_Suppl) was used. To achieve this dispensing profile, a cohort of SARS-CoV-2 positive gargle samples (n = 12), were inserted into the each of the four Hamilton STARlet’s loading racks at the 10th, 13th and 16th position (note that the Hamilton STARlet loads the plates vertically not horizontally). In the remaining positions, SARS-CoV-2 negative saline gargle samples (n = 80) were loaded (Fig. 3_Suppl).

### GDirect-PCR validation studies using Hamilton STARlet system

In the first phase of validation, saline gargle samples received at the BCCH’s Microbiology & Virology Laboratory for SARS-CoV-2 testing were tested on the HGDirect-PCR workflow using the SORP version: 2 assay (Fig. 4). These anonymized samples had been previously tested on the standard extraction assay (RdRp/E/RNaseP) (Fig. 4), and were tested on HGDirect-PCR assay within 12 h of receipt. The prospective testing was performed over 10 consecutive days, resulting in testing of 908 saline gargle samples.

In the second phase of the validation, non-anonymized saline gargle samples were tested prospectively by both the standard extraction PCR assay, and the HGDirect-PCR workflow. Interpretation and discrepant analysis were done prior to reporting, as described below.

### Time-motion studies

A time audit process was performed to determine the total hands-on time required to setup (a.) standard KingFisher RNA purification with manual pipetting and (b.) HGDirect-PCR for 92 samples and 4 controls. Each step of the process, including logging-in, barcode printing, sample aliquoting, and reagent preparation for extraction and PCR were individually timed (Fig. 4). The time audit process was done over three separate days, by three different medical laboratory technologists independently. The time taken for each step was averaged and rounded to the nearest minute.

### Interpretation algorithm of HGDirect-PCR results & discrepant analysis

A conservative interpretation algorithm for clinical samples was implemented in our laboratory to provide enhanced safety against contaminants and false-positives (Fig. 3). A sample was considered positive (“POS”) if both the SARS-CoV-2 genes—S and ORF8—were detected with C_T_ of ≤ 33. If both viral genes were undetected, the sample was considered negative (“NEG”), provided the human RNaseP gene was positive (C_T_ ≤ 50). Single SARS-CoV-2 target positive results (S^+^/ORF8^−^ or S^−^/ORF8^+^) were classified as “indeterminate” (“IND”), while those which failed to record any results for any of the gene targets (S/ORF8/RNaseP) were classified as “Invalid” (“IVLD”). If no sample was dispensed into the RT-qPCR assay reaction due to a liquid dispensing error, no RT-qPCR data were recorded and these samples were classified as “INVLD”. The IND/IVLD samples and weakly positive samples (C_T_ 33–40) were clinically resolved by testing the original patient sample on either the Xpert Xpress SARS-CoV-2 (Cepheid, Sunnyvale, CA, USA) or the BDMAX SARS-CoV-2 assay (Becton Dickinson, Quebec City, QC, Canada). For clinical reporting, the commercial testing result was accepted. The Xpert Xpress SARS-CoV-2 assay has been approved for the saline gargle sample type by Health Canada (https://www.canada.ca/en/health-canada/services/drugs-health-products/covid19-industry/medical-devices/authorized/expanded-use.html).

## Supplementary Information

Supplementary Figures.

## Data Availability

The datasets generated during and/or analyzed during the current study are available from the corresponding author on reasonable request.
