# Supplementary material for: Extraction-free clinical detection of SARS-CoV-2 virus from saline gargle samples using Hamilton STARlet liquid handler
Source: Sci Rep. 2023 Mar 14;13:4241. doi: 10.1038/s41598-023-30993-2 (PMC10013237; doi:10.1038/s41598-023-30993-2)
Supplement: Supplementary file 1 — Supplementary Figures. [file 41598_2023_30993_MOESM1_ESM.pptx]

## Slide 1
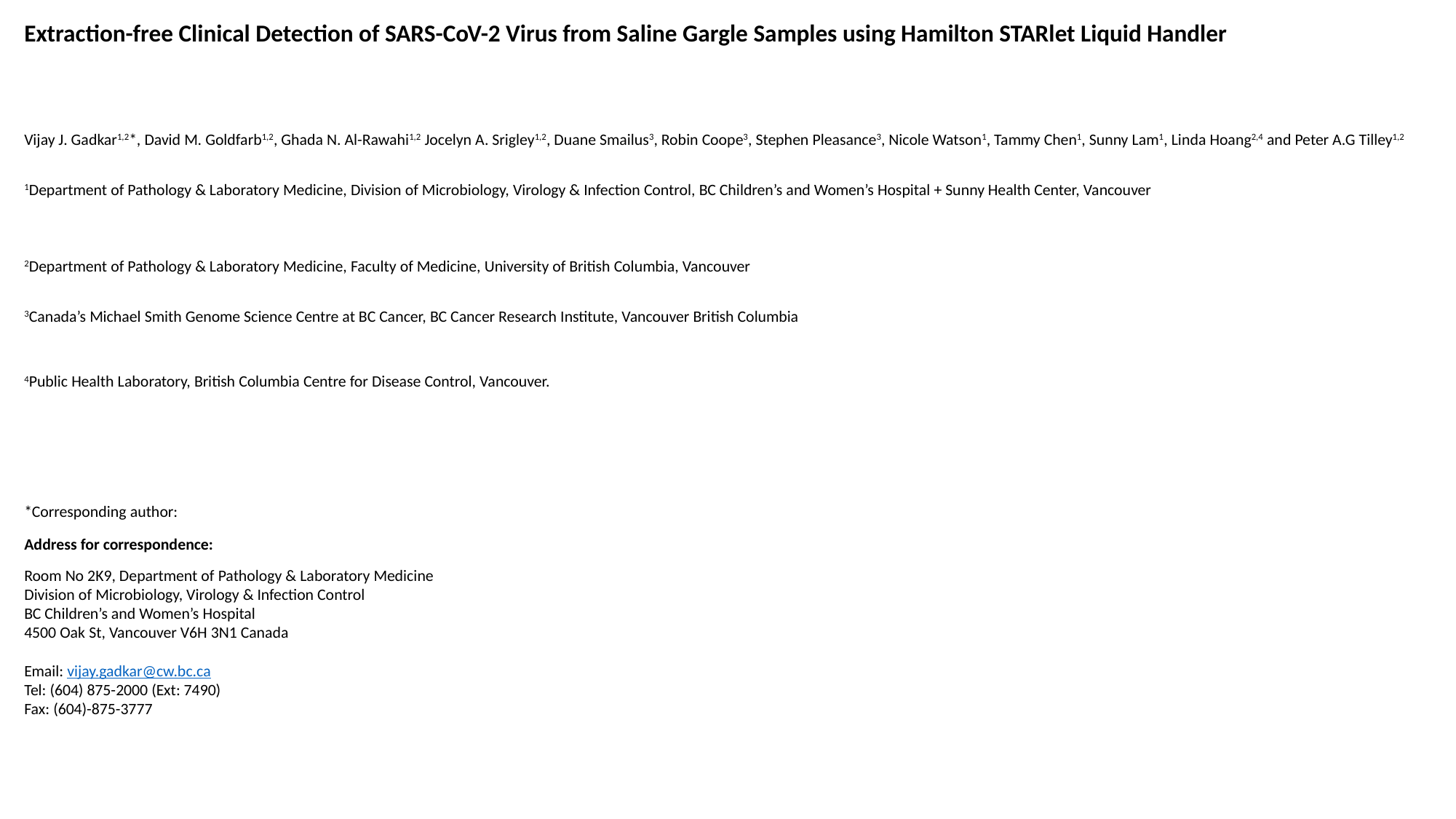

Extraction-free Clinical Detection of SARS-CoV-2 Virus from Saline Gargle Samples using Hamilton STARlet Liquid Handler
Vijay J. Gadkar1,2*, David M. Goldfarb1,2, Ghada N. Al-Rawahi1,2 Jocelyn A. Srigley1,2, Duane Smailus3, Robin Coope3, Stephen Pleasance3, Nicole Watson1, Tammy Chen1, Sunny Lam1, Linda Hoang2,4 and Peter A.G Tilley1,2
1Department of Pathology & Laboratory Medicine, Division of Microbiology, Virology & Infection Control, BC Children’s and Women’s Hospital + Sunny Health Center, Vancouver
2Department of Pathology & Laboratory Medicine, Faculty of Medicine, University of British Columbia, Vancouver
3Canada’s Michael Smith Genome Science Centre at BC Cancer, BC Cancer Research Institute, Vancouver British Columbia
4Public Health Laboratory, British Columbia Centre for Disease Control, Vancouver.
*Corresponding author:
Address for correspondence:
Room No 2K9, Department of Pathology & Laboratory Medicine
Division of Microbiology, Virology & Infection Control
BC Children’s and Women’s Hospital
4500 Oak St, Vancouver V6H 3N1 Canada
Email: vijay.gadkar@cw.bc.ca
Tel: (604) 875-2000 (Ext: 7490)
Fax: (604)-875-3777

## Slide 2
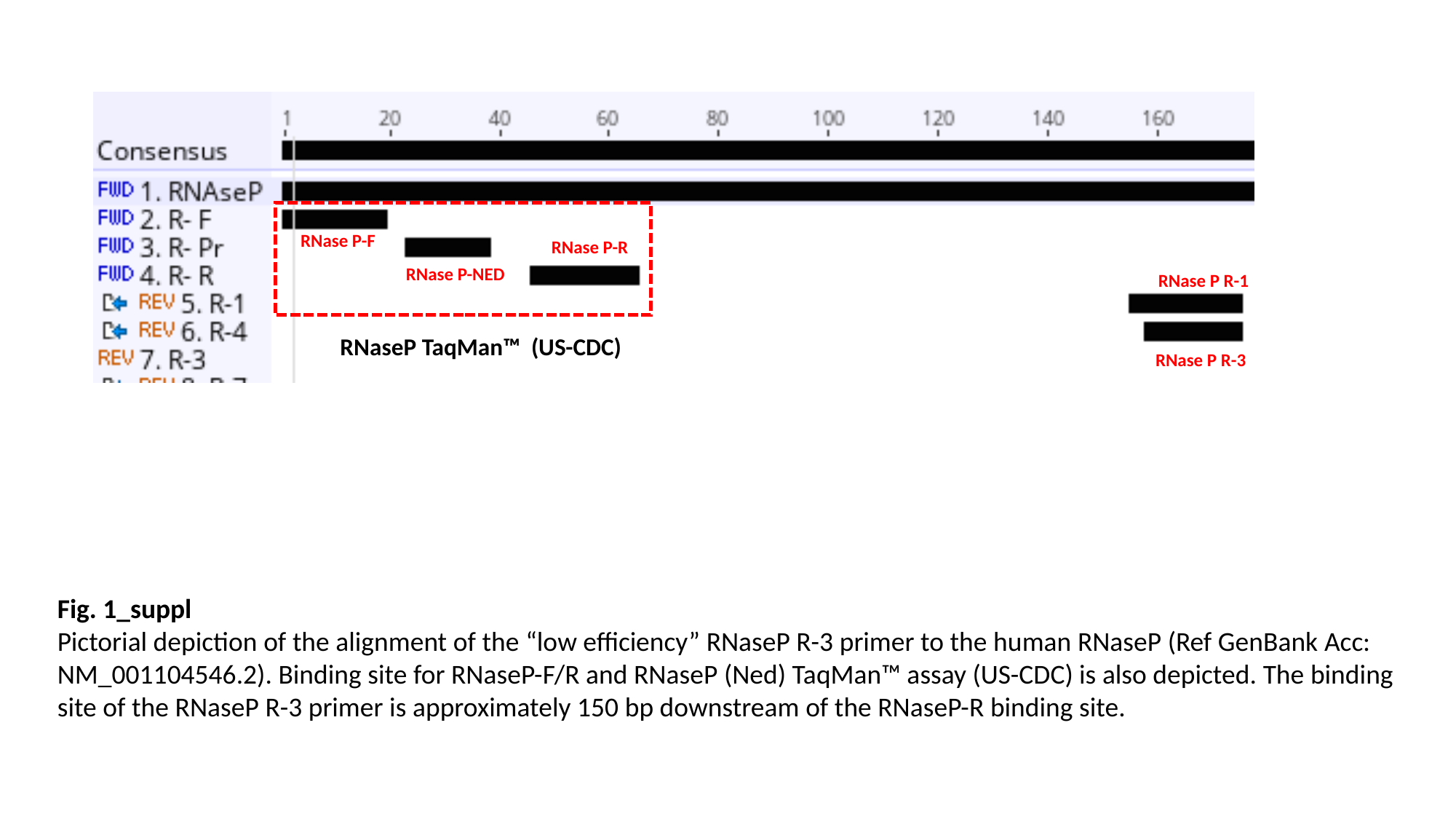

RNase P-R
RNase P-F
RNase P-NED
RNase P R-1
RNaseP TaqMan™ (US-CDC)
RNase P R-3
Fig. 1_suppl
Pictorial depiction of the alignment of the “low efficiency” RNaseP R-3 primer to the human RNaseP (Ref GenBank Acc: NM_001104546.2). Binding site for RNaseP-F/R and RNaseP (Ned) TaqMan™ assay (US-CDC) is also depicted. The binding
site of the RNaseP R-3 primer is approximately 150 bp downstream of the RNaseP-R binding site.

## Slide 3
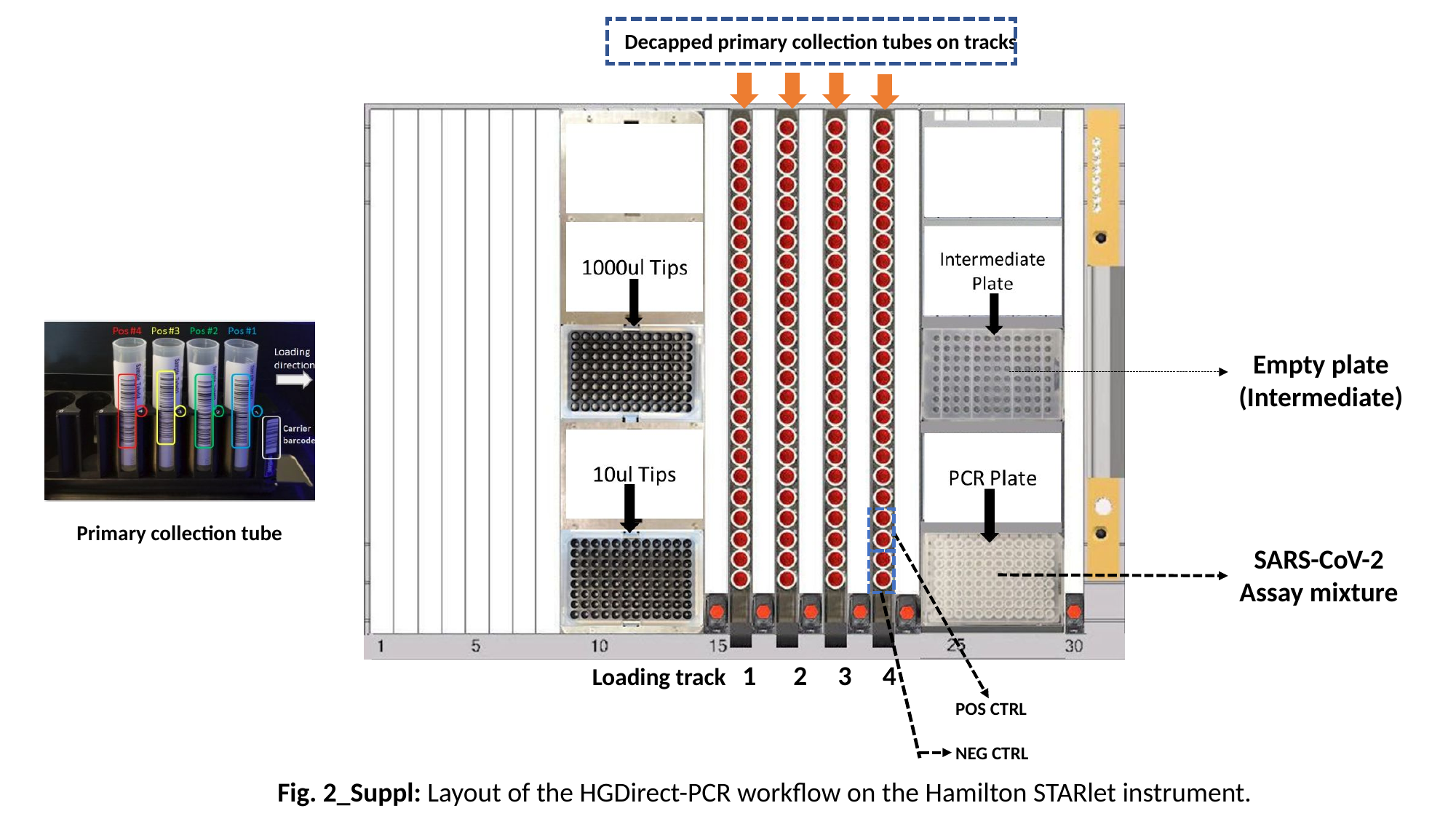

Decapped primary collection tubes on tracks
Primary collection tube
SARS-CoV-2
Assay mixture
Loading track 1 2 3 4
POS CTRL
NEG CTRL
Empty plate
(Intermediate)
Fig. 2_Suppl: Layout of the HGDirect-PCR workflow on the Hamilton STARlet instrument.

## Slide 4
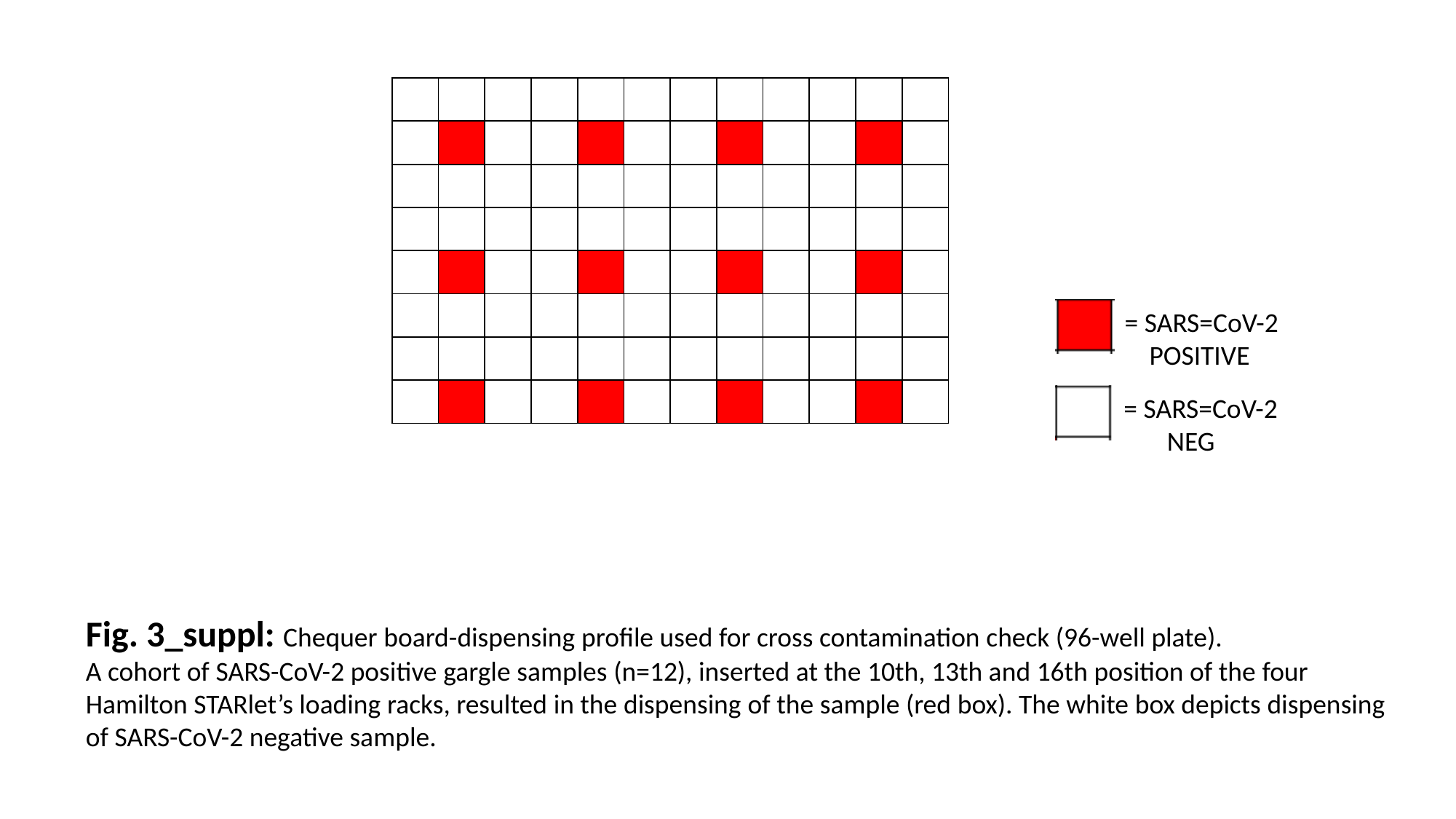

| | | | | | | | | | | | |
| --- | --- | --- | --- | --- | --- | --- | --- | --- | --- | --- | --- |
| | | | | | | | | | | | |
| | | | | | | | | | | | |
| | | | | | | | | | | | |
| | | | | | | | | | | | |
| | | | | | | | | | | | |
| | | | | | | | | | | | |
| | | | | | | | | | | | |
= SARS=CoV-2
 POSITIVE
= SARS=CoV-2
 NEG
Fig. 3_suppl: Chequer board-dispensing profile used for cross contamination check (96-well plate).
A cohort of SARS-CoV-2 positive gargle samples (n=12), inserted at the 10th, 13th and 16th position of the four
Hamilton STARlet’s loading racks, resulted in the dispensing of the sample (red box). The white box depicts dispensing
of SARS-CoV-2 negative sample.

## Slide 5
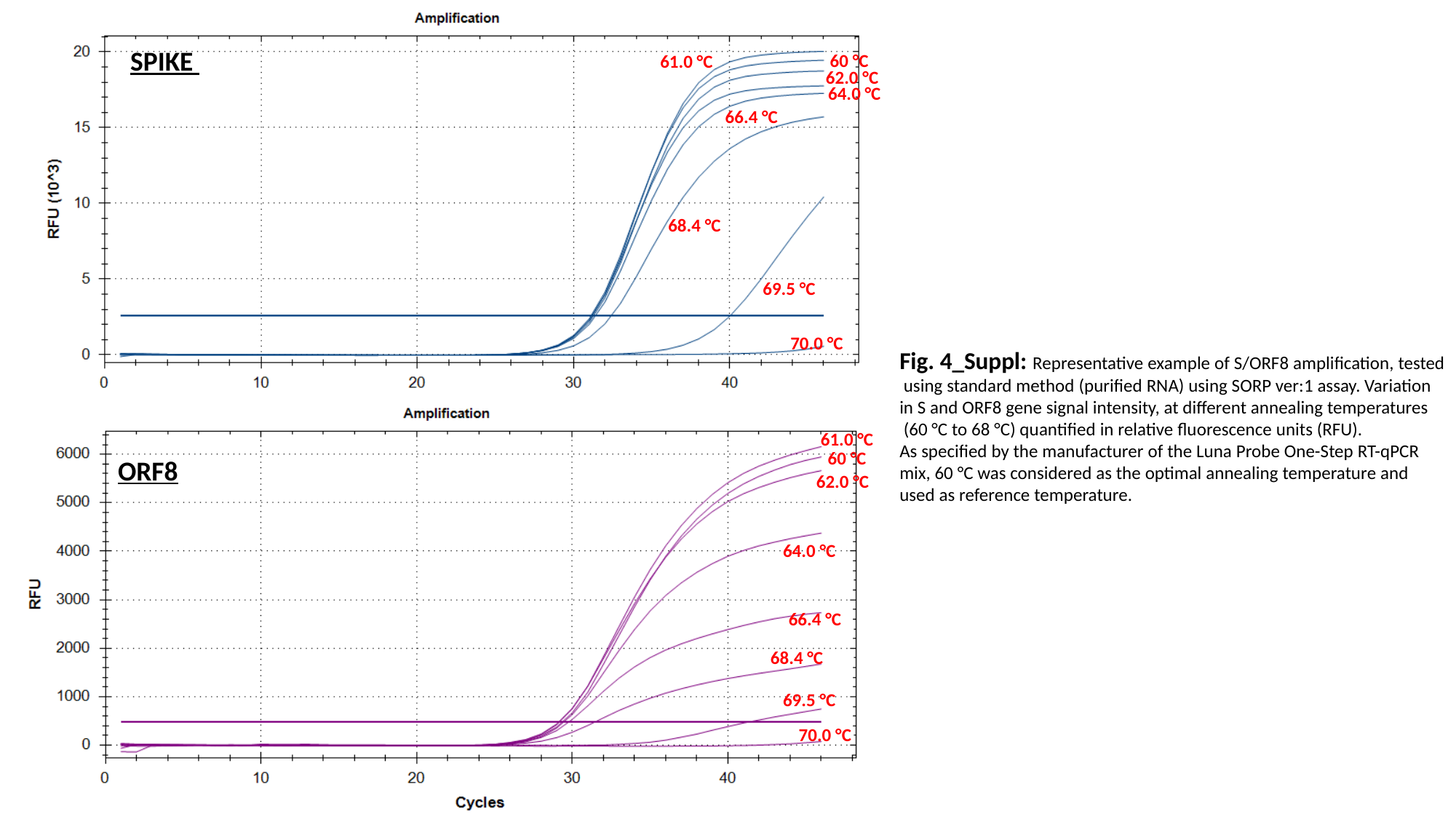

SPIKE
60 °C
61.0 °C
62.0 °C
64.0 °C
66.4 °C
68.4 °C
69.5 °C
70.0 °C
Fig. 4_Suppl: Representative example of S/ORF8 amplification, tested
 using standard method (purified RNA) using SORP ver:1 assay. Variation
in S and ORF8 gene signal intensity, at different annealing temperatures
 (60 °C to 68 °C) quantified in relative fluorescence units (RFU).
As specified by the manufacturer of the Luna Probe One-Step RT-qPCR
mix, 60 °C was considered as the optimal annealing temperature and
used as reference temperature.
61.0 °C
60 °C
ORF8
62.0 °C
64.0 °C
66.4 °C
68.4 °C
69.5 °C
70.0 °C

## Slide 6
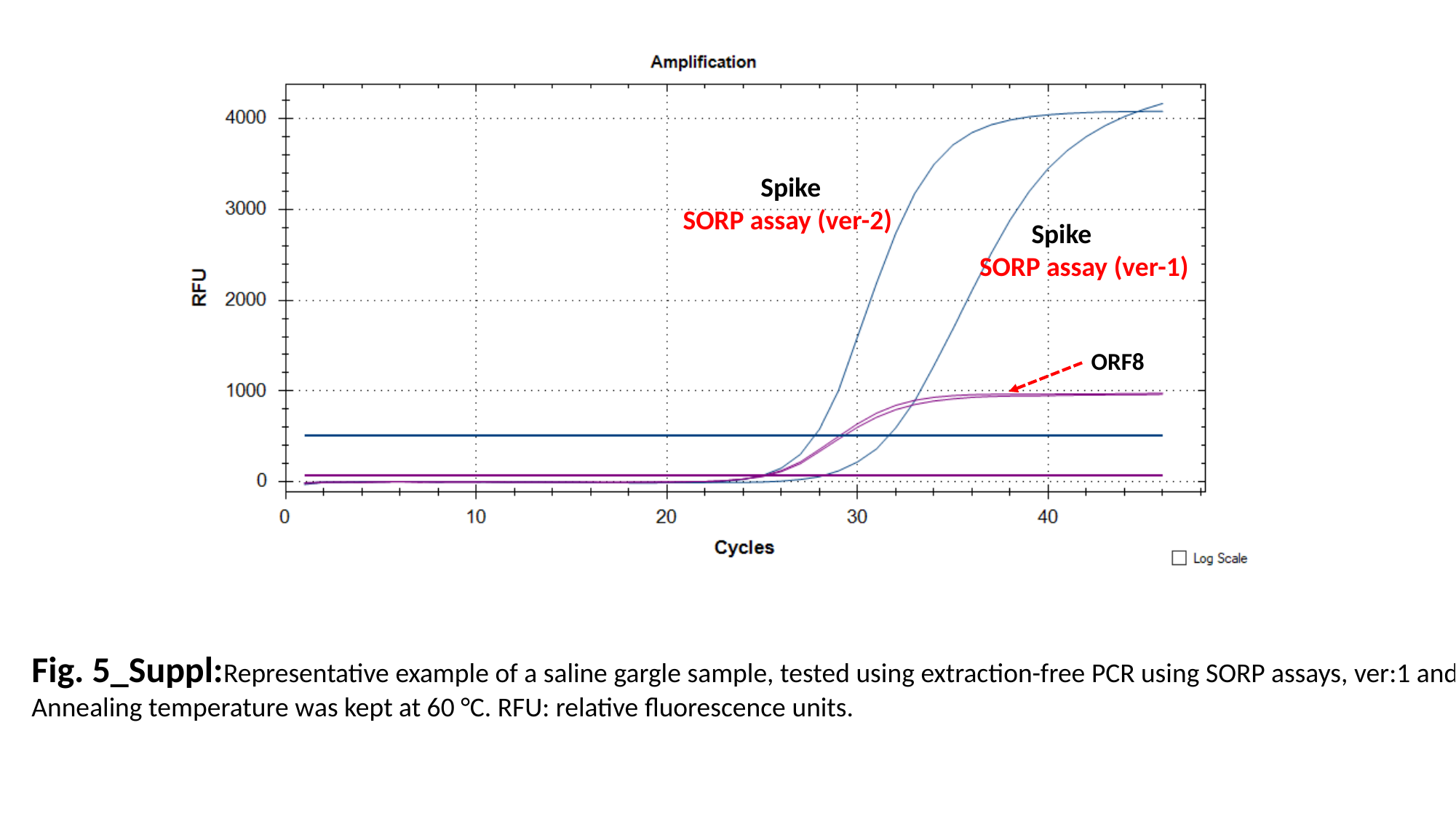

Spike
SORP assay (ver-2)
Spike
SORP assay (ver-1)
ORF8
Fig. 5_Suppl:Representative example of a saline gargle sample, tested using extraction-free PCR using SORP assays, ver:1 and 2.
Annealing temperature was kept at 60 °C. RFU: relative fluorescence units.

## Slide 7
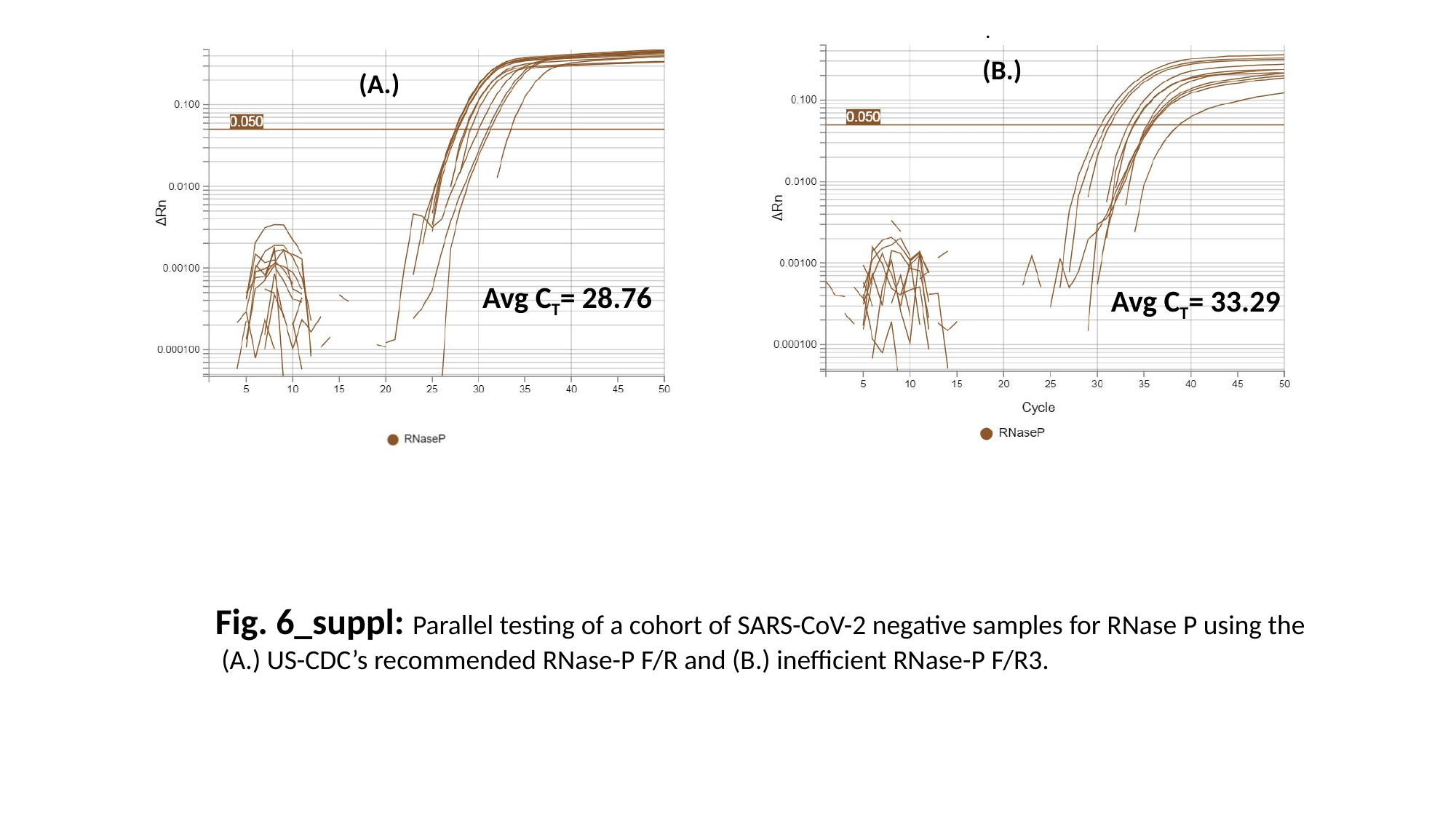

(B.)
Avg CT= 33.29
(A.)
 Avg CT= 28.76
Fig. 6_suppl: Parallel testing of a cohort of SARS-CoV-2 negative samples for RNase P using the
 (A.) US-CDC’s recommended RNase-P F/R and (B.) inefficient RNase-P F/R3.

## Slide 8
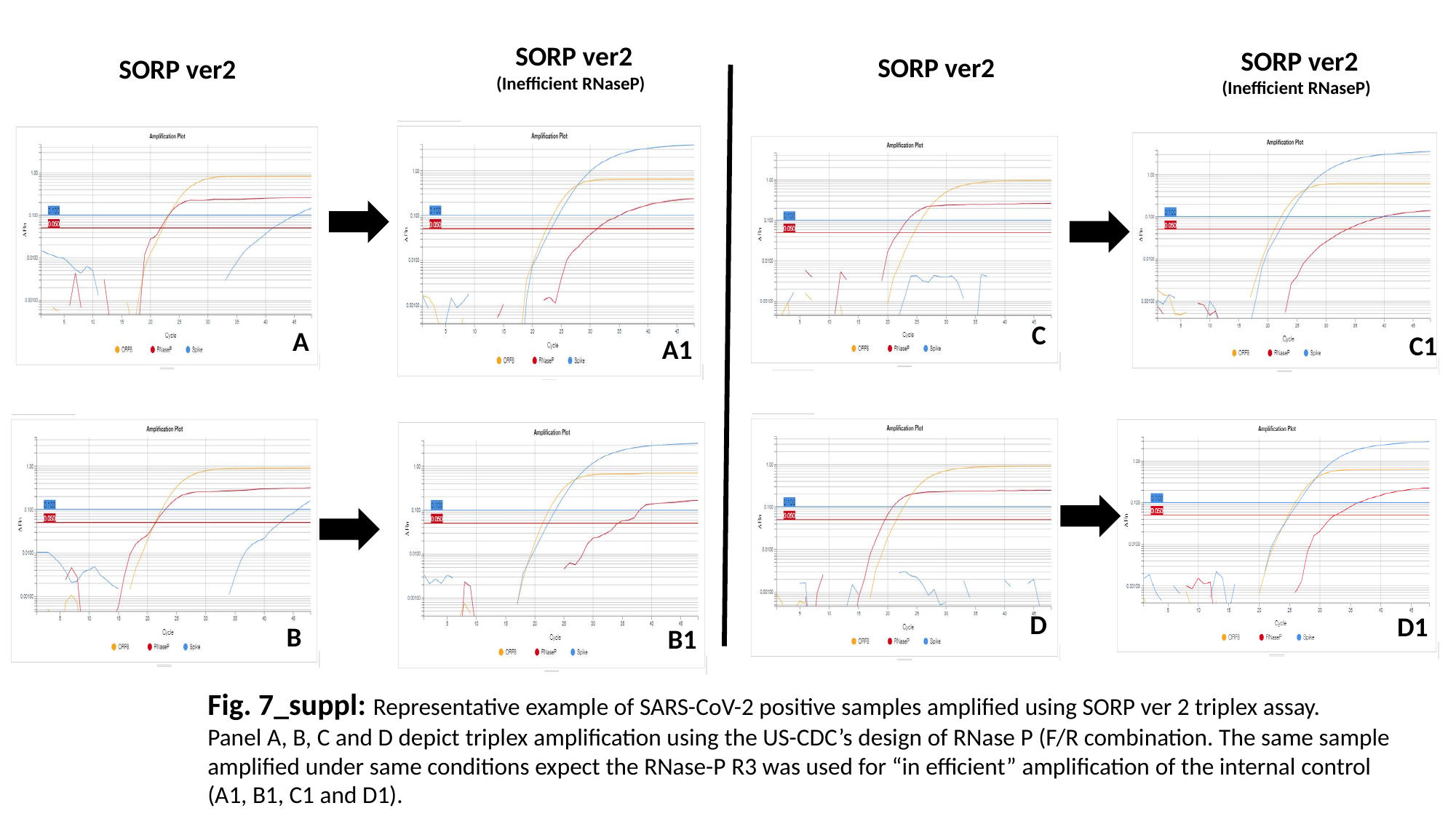

SORP ver2
(Inefficient RNaseP)
 SORP ver2
(Inefficient RNaseP)
SORP ver2
SORP ver2
C
A
C1
A1
D
D1
B
B1
Fig. 7_suppl: Representative example of SARS-CoV-2 positive samples amplified using SORP ver 2 triplex assay.
Panel A, B, C and D depict triplex amplification using the US-CDC’s design of RNase P (F/R combination. The same sample
amplified under same conditions expect the RNase-P R3 was used for “in efficient” amplification of the internal control
(A1, B1, C1 and D1).
